# China’s new cooperative medical scheme and equity in access to health care: evidence from a longitudinal household survey

**DOI:** 10.1186/1475-9276-12-20

**Published:** 2013-03-23

**Authors:** Wei Yang

**Affiliations:** 1 Department of Social Policy, LSE Health, The London School of Economics and Political Science, Houghton Street, London, WC2A 2AE, UK

**Keywords:** The NCMS, Insurance, Access to health care, Inequity, The rural population, China

## Abstract

**Introduction:**

China's New Cooperative Medical Scheme (NCMS) was brought to life in 2003 in response to the deterioration in access to health services in rural areas. Despite its fast expansion, the scheme’s impacts on access to health care have raised growing concerns, in particular regarding whether and to what extent the scheme has reduced inequity in access to health care in rural China.

**Methods:**

This study examines income-related inequity in access to health care from 2004 (before the national rollout of NCMS) to 2009 (after the expansion of NCMS across the rural China) by estimating Concentration Indices over both formal health care (outpatient care, prevention care) and informal health care use (folk doctor care). Data were drawn from a longitudinal household survey dataset - China Health and Nutrition Survey (CHNS).

**Results:**

The study suggested that the level of inequity remained the same for outpatient care, and an increased favouring-poor gap in terms of folk doctor care was observed. In terms of preventive care, a favouring-rich inequity was observed both in 2004 and 2009, but the effects of inequity were narrowed. The NCMS had some effects in reducing income-related health inequity in folk doctor care and preventive care, but the contribution was rather small. The study also found that the rural better-off had started to seek for commercial insurance to cover possible financial risks from the burden of diseases.

**Conclusion:**

The study concludes that the impacts of the NCMS on improving access to formal care for the poor are limited. Without a more comprehensive insurance package that effectively targets the rural poor, the intended equity goals expected from the scheme will be difficult to realize.

## Introduction

Countries across the world are looking to health insurance as a means of ensuring access to health care and protecting patients from financial risks. Health insurance has the potential to lower financial barriers of access to health care, since the financial risk of health care is shared among insurance participants and health cost will be reduced at the point of health care use [1]. One common way to organise insurance is to target its funds to either a group of the population, such as the vulnerable/disadvantaged socioeconomic population, or specific services that are most cost-effective and/or preferentially benefit the target population, such as primary care or outpatient care [1].

In China, a focus on health care for the rural population is gaining increased governmental attention in recent years. The government targets its public funds for health insurance by focusing on the rural population through the New Rural Cooperative Health Scheme (NCMS). Since “equitable access” has been officially declared by the State Council to be the principal aim of the rural health insurance reform [2], the main objective of the NCMS is to provide universal coverage and to improve equity and access to health care to the rural population regardless of individual characteristics such as job status, education, pre-existing condition, and level of wealth. For the past few decades, the state and enterprise funded health insurance only covered well-off urban employees, leaving the majority of the rural residents unprotected from health risks [3], the launch of the NCMS in 2003 is considered as a crucial step in closing the insurance gap and reducing inequity in access to health care for the rural population.

However, the real world experience actually tells us little about how far public health insurance can improve access to health care [3-7]. One major concern is whether the insurance is able to reach vulnerable/disadvantaged socioeconomic groups [3-5]. Evidence from developing countries also suggest that public voluntary insurance programmes, especially the ones that require substantial premiums and patient cost-sharing, may have little effect on improving the use of public financed health services of the poor. In Iran, despite the decent development of a few government health insurance schemes targeting the poor and catastrophic inpatient care in the last decades, co-payments still count for 58% of the health expenditures, and the proportion of people facing catastrophic health payment remained high even after the insurance reform [8]. In India, the newly developed insurance - Rashtriya Swasthya Beema Yojna(RSBY) which aimed to target the poor only allowed a limited rate of reimbursement for inpatient care. Studies found that expenditures on drugs claims which constituted around 75% of OOP payments and 80% of the spending on outpatient visits were not covered, and the impacts of the RSBY on protecting the poor against health payment-induced impoverishment were limited [9].

The launch of the New Rural Cooperative Medical Scheme (NCMS) in 2003 represents a major step of the Chinese government to move towards a more equitable and efficient rural health financing system, but it is not clear that the is sufficient enough to deliver equitable access in different types of health care. One major concern is that, under the NCMS, health care is provided in public health facilities through a fee-for-service reimbursement, and the reimbursement rates vary by different types of care and at different health facilities. Although the NCMS has extended its coverage to outpatient care since 2007, its emphasis is mainly on “catastrophic outpatient cost", and reimbursement is made either through participants' Medical Saving Account or pooled funds which requires substantial cost-sharing [10]. Further, the scheme only reimburses drugs listed on the National Essential Drug Reimbursement List, services covered by the insurance package, and care sought at state-owned public health facilities. The claimed reimbursement rates are the highest for care delivered at village/township health centres and the lowest at city/provincial hospitals, while care sought at the high level health facilities is usually associated with severe illness and high medical expenditures [11,12]. Consequently, as argued by many scholars, despite the broad coverage, co-payments for the NCMS participants in general remained high even after the insurance claims were made, and this may impede a subpopulation of the rural poor from seeking care [13-16]. In terms of outpatient care, scholars argued that the NCMS increased the use of outpatient care among the poor at village clinics, whereas the increased use in inpatient care overall and at the higher level health facilities was concentrated disproportionately only among people who were rich [17]. Liu et al. [3] and Yu et al. [18] also found that the NCMS only increased the use of inpatient care for the better-off, whereas it had no significant impact on outpatient use.

While the previous work is important, the investigation on how the NCMS impacts inequity in health use is subjected to little updated empirical research, which is the setting for this paper. Previous studies either limit their investigations to a given point in time [15,17,19], or a specific health service [16]. The NCMS has been implemented for a few years, but it is still not clear if the scheme has any impact on utilisation. If it were, as the reimbursement rates of the NCMS are set at different levels for different services, it is worth investigating how much it may influence the utilisation for different socioeconomic groups, and whether variations in use is a generalised phenomenon, or are observed only for some services. For instance, little information is available on the characteristics of the users of preventive care and folk doctors, consequently, how the NCMS impacts the use.

Another issue that is largely neglected in previous studies, but is central to the measurements of equity in use is that studies tend to overlook the association between health use and health needs. Inequity in health use may be driven by factors as legitimate/fair inequity, such as health needs, and illegitimate/unfair inequity that arise from circumstances beyond individual’s control, such as per capita income. Policy may concern less with legitimate inequities because inequity arising from legitimate factors, e.g. health needs, is usually reasonable and acceptable. Therefore, a measure of socioeconomic related health inequality should control for legitimate differences, in this case, factors associated with health needs [20].

To shed light to above issues, this paper measures the extent to which the NCMS affects health care utilisation on the rural population in China, considering two types of formal health care (outpatient care and prevention care), and one type of informal health care (folk doctor care [1]). This paper first compares the magnitude of inequities in health use in 2004 (before the national rollout of NCMS) and 2009 (after the expansion of NCMS across the rural China). The Concentration Indices for utilisation, which compares the cumulative distribution of health use with the cumulative distribution of the population ranked by individual wealth, is used [21,22]. It then investigates the determinants of patterns of health care use and the characteristics of the users for different services, taking into account the contribution of the NCMS to equity in health use. Data are drawn from China Health and Nutrition Survey 2004 and 2009.

The empirical findings derived from the study are expected to feed back into the policy making process. In particular, we are concerned that the expansion of the NCMS does not necessarily lead to equal access to care. Drawing from the literatures discussed above, this paper identifies situations in which at least one of them is expected to dominate. First, the launch of the NCMS is a means of improving the equitable access to formal care and discouraging the use of informal care/folk doctor care. Folk doctor care is not covered by the insurance scheme, whereas outpatient and inpatient care are included in the insurance package. Since the reimbursement rate set for formal care is relatively low, co-payments is likely to become one of the barriers to impede access to formal care among the poor. The NCMS may have positive impacts in reducing the use of informal care; however, the impact may be limited since unmet health need may still lead to increased use of informal care, which is less costly and widely accessible compared with formal care. Second, the NCMS may also exert some positive influence on use of preventive care, which historically required more cost-sharing and is now partially covered by the NCMS (e.g. general physical examination, blood pressure screening, and prenatal examination). As the co-payments are still high, preventive care use may still appear concentrated among the rich, but the level of inequity may become less pronounced.

In the subsequent sections, a background of the NCMS is provided; followed by methods, results, and a discussion of the policy implications.

### The New rural cooperative medical scheme

In rural China, a traditional rural cooperative medical insurance scheme was established in the 1950s. There was no real premium transfer in the rural system. Such a scheme was based on the People’s Commune system [2]. Folk doctors provided primary care free of charge to the rural population most of the time, although individuals occasionally needed to pay a limited amount of out-of-pocket fee for medicines [23].

Following the collapse of the People’s Commune System in the early 1980s, the Cooperative Medical Scheme (CMS) virtually disappeared. Within two decades, the proportion of rural residents covered by health insurance shrank from 90% at the end of the 1970s to less than 5% in the late 1990s, and rural residents were on their own in paying for health care (Ooi, 2005). The burden of disease was exacerbating poverty across the rural China - average per episode inpatient cost in rural areas had increased from 613RMB (US$98.35) in 1993 to 2,649RMB (US$425.02) in 2003, and the percentage of rural populations did not seek care when recommended was 63.7% in 1998 and 75.4% in 2003 [2,24].

In response to the dire need for affordable health care, the New Cooperative Medical Scheme (NCMS) was brought to life in China in 2003. The scheme was initially proposed by the National Rural Health Conference in 1996, and formally adopted during the 16th National Congress of the Communist Party of China in 2002 [3,25]. According to the scheme, the premium was largely subsidized by local and central government, and individual’s contribution to the premium was relatively low. In the western and central China, where the level of economic development was low, the central government assisted with the local government to provide financing sources for the scheme. In the more affluent eastern and coastal region, financing the premium was mainly through local government. According to the official government statistics, from 2003 to 2008, the coverage of the scheme expanded dramatically. By the end of 2008, 726 million rural residents in 2448 counties were covered by the scheme [2]. According to the 2012 Report on the Work of the Chinese Government, the scheme had covered 832 million rural residents, or 97.5% of Chinese farmers by 2012; government contribution to insurance premium increased from 10 RMB(US$1.60) in 2003 to 240 RMB(US$38.51) in 2012; Insurance packaged had expanded from covering mainly catastrophic illness to outpatient and prevention care [26]. The Chinese Health Minister Chen Zhu regarded NCMS as one of the largest medical security scheme in the world [27]. Table 1 shows the main characteristics of the NCMS.

**Table 1:** Features of the NCMS

	**NCMS**
Date started	2003 (Pilot scheme was initiated in four provinces)
Enrollment	Voluntary at household level
Coverage	94.3% in 2009
Guideline	General guidelines are issued by the central government, local governments retain considerable discretion over the details
Administration	County government sets the reimbursement rate, ceilings, medical saving account, etc.
Risk pooling	County level
Target population	Rural residents (840 million)
Financing mechanism	In the western and central China, the central government assisted the local government in providing financing for the scheme. In the more affluent eastern and coastal region, financing the premium was mainly through local government.
Designated health facilities	All levels of health facilities
Covered services	Inpatient series, catastrophic outpatient services, some prevention care services

## Methods

Income-related inequity in health is estimated by pooled Probit Model and well-established methods based on the Concentration Indices [21,28]. The method involves three basic steps: (1) estimate pooled Probit Models on the determinants of health use, and predict need (indirectly) standardized health for each health variable, and for each year separately; the computation of variance inflation factors (VIF) indicates that multicollinearity is not a problem. Ramsy RESET tests are performed, and results show the models have no specification problems; (2) calculate the Concentration Indices for actual use EI (the inequity driven by the actual health care utilisation), the horizontal equity indices HI (the inequity driven by socioeconomic factors); (3) decompose the socioeconomic factors that contribute to the inequities for the each year to see whether contributions have changed over time.

The multivariate regression models of health variables for step (1) and (2) above are central to the methods. The nature of the health use variables (binary variables) formally calls for a non-linear estimation. However, the disadvantage of this procedure is that the certain components of the equity analysis, such as decomposition analysis are difficult to implement and interpret. Studies have shown that equity measurements calculated by Linear Probability Model (LPM) do not differ importantly from the non-linear estimation [28,29]. Therefore, the paper will use LPM instead of non-linear regression to standardize the health variables and to decompose the Concentration Indices. Results from Probit Model are nonetheless presented in the appendices for comparisons. Instead of using the Concentration Indices, Erreygers’s Concentration Index, which has been recently developed and has proved to be a better estimation for binary variable, will be used [30-33].

### Statistical analysis

#### Need standardization

The standardized health (*ŷ*_*i*_^*X*^) is obtained by a regression of actual health use (*ŷ*_*i*_) as the following:

(1) $$y_{i}=\alpha +\sum_{j}\beta_{j}x_{\text{ji}}+\sum_{k}\gamma_{k}z_{\text{zi}}+\varepsilon_{i}$$

Where *x*_*j*_ are the health need variables, i.e., age, sex and health needs, *Z*_*k*_ are non-need/socioeconomic variables, i.e., (the logarithm of) income, education, job status, provinces of residence, urban/rural,,marital status, *α,β,* and *γ* are the parameter vectors, and *ε* is the error term.

The coefficients from Linear Probability Model estimations are obtained with actual values of the *x*_*j*_ variables, i.e. health needs, that are to be standardized for, and the sample mean for *Z*_*k*_ variables that are not to be standardized but to be controlled for. The predicted values of the health indicator ${\hat{y}}_{i}^{X}$ are then obtained.

(2) $${\hat{y}}_{i}^{X}=\hat{\alpha }+\sum_{j}{\hat{\beta }}_{j}x_{\text{ji}}+\sum_{k}{\hat{\gamma }}_{k}{\bar{z}}_{\mathrm{zi}}$$

Assuming a linear model, estimates of indirectly standardized health, *ŷ*_*i*_^*IS*^ can be obtained by calculating the difference between actual health (*y*_*i*_) and standardized health (*ŷ*_*i*_^*X*^), plus the sample mean ( $\bar{y}$)

(3) $${\hat{y}}_{i}{}^{\text{IS}}=y_{i}-{\hat{y}}_{i}^{X}+\bar{y}$$

Rearranging the equation (2.3),

(4) $${\hat{y}}_{i}{}^{\text{IS}}=y_{i}-\sum_{j}{\hat{\beta }}_{j}\left(x_{\text{ji}}-{\bar{x}}_{j}\right)$$

Equation (4) shows that the standardization is to subtract the variation of health use driven by health need factors from actual health use. Therefore, the distribution of ${\hat{y}}^{\text{IS}}$ across income can be interpreted as the health use that an individual would expect to be observed, irrespective of differences in the distribution of the characteristics associated with health needs.

#### Concentration indices

The Concentration Index has been used in many studies to quantify the degree of socioeconomic related inequality in health variables [22,34-36]. Concentration indices quantify the degree of socioeconomic related inequality in a health variable. There are many ways to express the Concentration Index, however, the one utilised here is:

(5) $$\text{CI}=\frac{2}{\mu }\mathrm{cov}\left(h_{\text{it}},R_{i}^{t}\right)$$

Where *i* represents the individual, *h*_*i*_ is the health variable, R is the individual’s living standard rank, μ is the mean of the health variable in the population, and t is the year. If there is no socioeconomic-related inequality, the index is zero. A positive value indicates a pro-rich inequality, and a negative value indicates a pro-poor inequality.

However, recent studies suggest there are some limitations to the Concentration Index. Wagstaff [37] has found that if the health variable of interest is binary (thus takes the value of 0 or 1), then the bounds of the Concentration Index depend on the mean of the health variable. The bounds turn out to be wider for populations with a low mean (i.e. close to 0) than for populations with a high mean (i.e. close to 1) [30,31]. Therefore, this paper uses Erreygers’s Concentration Index [30], which is recently introduced to take the above concerns this into account:

(6) $$E\left(h\right)=\frac{4\mu }{\left(b_{n}-a_{n}\right)}C\left(h\right)$$

Where b_n_ and a_n_ represent the maximum and minimum of the health variable (*h*), *μ* is the mean of the health variable in the population, and *C* (*h*) represents the Concentration Index specified in (5).

The range of the Erreygers’s Concentration Index is from −1 to 1. A positive value indicates pro-rich inequality, meaning that health use is more concentrated among the better-off. A negative value indicates pro-poor inequality, meaning that health service is more concentrated among the poor.

The study also provides variance estimates or confidence intervals. Confidence intervals were calculated using bootstrapping methods [38,39]. The numbers of replication were set at 1000.

#### Decomposition analysis

Decomposition analysis helps to capture the contribution of each individual factor to income-related health inequality [21:159, 40].

The decomposition of Erreygers’s Concentration Index is carried out by transforming the health variable *h*_*i*_ = (*h*_*i*_ − *a*_*h*_)/(*b*_*h*_ − *a*_*h*_)*.* Therefore, the Erreygers’s index differs from the decomposition of *C* by the multiplication by 4 and *μ*_*h*_. The equation is as follows.

(7) $$E=4\left[\beta \mu_{y}C_{y}+\sum_{j}\gamma_{j}\mu_{\text{zj}}C_{\text{zj}}+\sum_{k}\delta_{k}\mu_{\text{xk}}C_{\text{xk}}\right]$$

Where μ is the mean, *j* represents a vector of a set of variables *z*_*j*_, *k* represents a vector of variables *x*_*k*_, *γ* represents the coefficient of the variable z, *δ* represents the coefficient of the variable x, *C* is the Concentration Index for x.

Another critical problem arises from calculation of the Concentration Index is the ranking indicator of the income measurements. Studies have found that repetitive values of the ranking variables, i.e. two of more observations have the same values of the living standard variables, may bring instability for the calculation [40,41]. In this paper, we have sorted the data both in ascending and descending order to test the consistency of the Erreygers’s Index, and to obtain the boundaries of Erreygers’s Index. Results suggest that no change is observed in terms of the value of the indices.

### Data source and variable specification

CHNS, the data source for the paper, is an ongoing open cohort, international collaborative project between the Carolina Population Center at the University of North Carolina at Chapel Hill and the National Institute of Nutrition and Food Safety at the Chinese Center for Disease Control and Preventions. Data from CHNS 2004 and 2009 are used. A multistage, random cluster sampling process was used to draw the sample in nine provinces in China, e.g. Liaoning, Heilongjiang, Jiangsu, Shandong, Henan, Hubei, Hunan, Guangxi, and Guizhou. Counties in the nine provinces were stratified by income (low, middle, and high), and a weighted sampling scheme was used to randomly select four counties in each province. In addition, the provincial capital and a lower income city were selected when feasible. Villages and townships within the counties and urban and suburban neighbourhoods within the cities were selected randomly. In the most recent survey conducted in 2009, a total of 4400 households with a total of 26,000 individuals were included in the sample. The survey was designed to examine the effects of the health, nutrition, and family planning policies and programs implemented by national and local governments and to see how the social and economic transformation of Chinese society is affecting the health and nutritional status of its population.

CHNS is a representative sample for population dwelling in the surveyed provinces. The rural sample totals 5,361 observations in 2004 and 5,232 observations in 2009. The analysis included 4,351 observations in 2004 and 3,919 observations in 2009 after dropping observations under 18 and with missing data.

#### Dependent variables

Formal (outpatient care, prevention care) and informal health care use (folk doctor care) are analysed for the likelihood of a visit (no visits versus one or more visits). Specific questions are as follows: for outpatient care variable, respondents were asked: “Have you sought outpatient care during the past 4 weeks? 0 No, 1 Yes, and 9 Unknown”. For the prevention care variable, respondents were asked: “During the past 4 weeks, did you receive any preventive health service, such as health examination, eye examination, blood test, blood pressure screening, tumour screening? 0 No, 1 Yes, and 9 Unknown”. For the folk doctor care variable, respondents were asked: “Did you visit a folk doctor last year? 0 No, 1 Yes, and 9 Unknown”.

#### Independent variables

Per capita income data are used as the measurement of living standard. Although using household expenditure as a measurement of living standard are suggested in a number of studies on health equity in developing countries [42,43]. Scholars argue that household expenditure may not be a reliable indicator for living standard measurement in the context of China [44]. China has the highest saving rate in the world; expenditure data are distorted by the propensity to save for emergencies and thus may not be a good proxy as living standard indicators [45-47]. Therefore, this study uses income variables instead of expenditure variable as the indicator for living standard measurements. Household income data are measured as gross annual household income aggregated from all sources including: gardening, farming, livestock/poultry, fishing, handicraft and small commercial household business inflated to 2009 (the last wave of the survey). As this paper examines individual level of health care use, it is important to adjust household estimates of aggregate income to reflect household size and composition. This is done by Equivalence Scale, which is constructed as some function of the household size and demographic composition provided estimates are available for household economies of scale and the cost of children: *AE* = (*A* + *αK*)^*θ*^[48]. *A* represents the number of adults in the household, Κ represents the number of children, α is the “costs of children”, and *θ* is the degree of economies of scale. The value of α should be high when most goods are private and low when most of the household expenditure is on shared goods. A value of 0.75 to 1.0 is suggested when food expenditures account for a large proportion of total household income, which means that the economies of scale are limited [21:77]. In this paper, α is set as 0.3, and *θ* is set as 0.75.

Need variables are age, split into four categories (18 to 29, 30 to 44, 45 to 64, and 65 and above), gender, and morbidity types split into two categories (major illness, minor illness and others) [28,49]. Need variables are also measured by asking whether the respondent has been ill or injured during the past 4 weeks.

Non-need/socioeconomic variables included are education, occupations, marital status, insurance types, urban/rural residency, and provinces of residency. Education is categorized into four groups: no education, primary and secondary education, high school and technical school education, and university education and above. University education and above is used as the reference group. Occupations are categorized into four groups: white collars/professionals, unskilled workers/agricultures, unemployed, and other. For the province variable, province Guizhou is set as the reference group. Whether the respondent is classed as urban or rural is based on his/her registration status as on his/her Hukou [3] booklet. Finally, insurance coverage is included as a non-need/socioeconomic variable.

A summary of dependent and independent variables are listed in Table 2.

**Table 2:** Descriptive statistics for the study population (mean/standard deviation)

		**2004(N = 4351)**		**2009(N = 3919)**	
**Variable**	**Definition**	**Mean**	**S.D.**	**Mean**	**S.D.**
***Health use variables***					
Outpatient use	Dummy variable: 1, outpatient use; 0 otherwise	0.111	0.314	0.116	0.320
Folk doctor use	Dummy variable: 1, folk doctor use; 0 otherwise	0.033	0.179	0.050	0.218
Preventive care use	Dummy variable: 1, Preventive care use; 0 otherwise	0.030	0.170	0.035	0.184
***Health needs variables***					
18-29	Dummy variable: 1, aged between 18–29; 0 otherwise.	0.122	0.328	0.098	0.297
30-44	Dummy variable: 1, aged between 30–44; 0 otherwise.	0.333	0.471	0.310	0.462
45-64	Dummy variable: 1, aged between 45–64; 0 otherwise.	0.447	0.497	0.469	0.499
65 and above	Dummy variable: 1, aged between 65 and above; 0 otherwise.	0.097	0.296	0.124	0.329
Gender	Dummy variable: 1, male; 0 female	0.499	0.500	0.506	0.500
No symptoms	Dummy variable: 1, no symtons; 0 otherwise	0.784	0.412	0.801	0.400
Minor Illness	Dummy variable: 1, minor illness; 0 otherwise	0.152	0.359	0.137	0.344
Major illness	Dummy variable: 1, major illness; 0 otherwise	0.064	0.245	0.062	0.242
4 week illness	Dummy variable: 1, having been illness for the past 4 weeks; 0 otherwise	0.153	0.360	0.148	0.355
***Socioeconomic variables***					
Per capita income	Per capita household income inflated to 2009	4787.057	5004.990	9996.772	11817.190
No insurance	Dummy variable: 1, no insurance; 0 otherwise	0.888	0.315	0.067	0.250
NCMS	Dummy variable: 1, NCMS; 0 otherwise	0.041	0.197	0.875	0.331
Commercial insurance	Dummy variable: 1, commercial insurance; 0 otherwise	0.013	0.112	0.029	0.167
Other insurance	Dummy variable: 1, other insurance; 0 otherwise	0.059	0.235	0.030	0.170
Marital Status	Dummy variable: 1 married, 0 otherwise	0.874	0.332	0.883	0.321
White collar/skilled	Dummy variable: 1 white collar or skilled worker, 0 otherwise	0.065	0.246	0.072	0.258
Unskilled/farmer	Dummy variable: 1 unskilled worker or farmer, 0 otherwise	0.617	0.486	0.691	0.462
Other job	Dummy variable: 1 other jobs, 0 otherwise	0.021	0.143	0.029	0.169
Unemployed	Dummy variable: 1 Unemployed, 0 otherwise	0.225	0.418	0.207	0.405
No edu	Dummy variable: 1 no education; 0 otherwise	0.216	0.412	0.240	0.427
Pri and sec edu	Dummy variable: 1 primary and secondary education; 0 otherwise	0.628	0.483	0.604	0.489
High school	Dummy variable: 1 high school and technical school education; 0 otherwise	0.139	0.346	0.128	0.334
Uni and above	Dummy variable: 1 university education and above; 0 otherwise	0.017	0.129	0.028	0.164
Province Liaoning	Dummy variable: 1 Liaoning, 0 otherwise	0.123	0.329	0.119	0.323
Province Heilongjiang	Dummy variable: 1 Heilongjiang, 0 otherwise	0.099	0.299	0.110	0.312
Province Jiangsu	Dummy variable: 1 Jiangsu, 0 otherwise	0.126	0.332	0.121	0.326
Province Shandong	Dummy variable: 1 Shandong, 0 otherwise	0.108	0.310	0.111	0.315
Province Henan	Dummy variable: 1 Henan, 0 otherwise	0.099	0.298	0.100	0.300
Province Hubei	Dummy variable: 1 Hubei, 0 otherwise	0.103	0.304	0.107	0.310
Province Hunan	Dummy variable: 1 Hunan, 0 otherwise	0.085	0.279	0.090	0.286
Province Guangxi	Dummy variable: 1 Guangxi, 0 otherwise	0.124	0.330	0.133	0.339
Province Guizhou	Dummy variable: 1 Guizhou, 0 otherwise	0.132	0.339	0.110	0.312

## Empirical results

### Descriptive statistics

Some differences in health care use are observed across years. Table 2 compares the share of health care use by years. Results show that the use of outpatient care remained the same between 2004 and 2009, while the use of folk doctor care had increased and the use of preventive care had decreased.

A significant increase in insurance coverage was observed. In 2004, 88.8% of the rural Chinese were not covered by any insurance; the percentage decreased to 6.7% in 2009. In the meantime, a significant increase in terms of the coverage of NCMS was observed from 2004 to 2009. In 2004, only 4.0% of the rural Chinese were covered by NCMS, the percentage increased to 87.5% in 2009. The probability of participating in commercial insurance also increased from 1.3% to 2.9%. Those who reported to be covered by “other insurance” were most likely to be retired military or government officials, and family members of deceased military and/or government officials. These reimbursement rates for these insurances were much higher compared with other public health insurances in China.

### Determinats of individual health care use

Table 3 presents the estimations of the determinants: the maximum-liklihood marginal effects of Probit Model.

**Table 3:** Determinants of health service use (probit model)

	**Outpatient**		**Folk doctor**		**Preventive care**	
	**Coefficient**	**S.E.**	**Coefficient**	**S.E.**	**Coefficient**	**S.E.**
**Need variables**						
Age group (ref = 18–29)						
30-44	−0.036	0.128	0.328***	0.115	−0.097	0.381
45-64	0.043	0.123	0.285**	0.114	−0.182	0.1
65 and above	0.116	0.144	0.352***	0.134	−0.123	0.399
Gender (1 = male)	−0.115*	0.063	−0.009	0.055	−0.147**	0.026
Morbidity types (ref = Major illness)						
No symptoms	−2.373***	0.109	−0.487***	0.118	−0.658***	0
Minor Illness	0.02	0.073	−0.091	0.097	−0.282***	0.009
4 week illness	0.715***	0.068	0.183*	0.096	0.149	0.179
**Non-need/socioeconomic variables**						
Per capita income (lg)	0.049	0.037	−0.009	0.034	0.087**	0.033
Insurance type (ref = no insurance)						
NCMS	−0.021	0.118	−0.199*	0.107	0.163	0.152
Commercial insurance	−0.511**	0.225	−0.216	0.234	0.413**	0.017
Other insurance	−0.024	0.139	−0.378**	0.17	0.48***	0
Marital Status (1 = married)	−0.033	0.092	0.113	0.089	0.084	0.407
Occupation (ref = white collar and skilled worker)						
Unskilled and agriculture	−0.158	0.119	0.123	0.125	−0.198*	0.073
Other job	−0.091	0.229	0.01	0.218	−0.189	0.388
Unemployed	−0.217*	0.126	0.211	0.131	−0.008	0.945
Education level (ref = uni and above)						
No edu	0.033	0.254	0.569	0.422	−0.568***	0.004
Pri and sec edu	0.057	0.244	0.472	0.418	−0.491***	0.006
High school	−0.145	0.247	0.502	0.42	−0.504***	0.005
Region (ref = Province Guizhou)						
Province Liaoning	−0.236*	0.122	−0.468***	0.115	0.159	0.288
Province Heilongjiang	−0.222	0.147	−0.904***	0.168	−0.247	0.199
Province Jiangsu	0.009	0.133	−0.424***	0.132	0.605***	0
Province Shandong	0.178	0.137	−0.084	0.105	0.61***	0
Province Henan	0.333***	0.119	0.108	0.09	0.151	0.327
Province Hubei	0.082	0.12	−0.506***	0.115	0.489***	0
Province Hunan	−0.199	0.136	−0.137	0.107	−0.03	0.863
Province Guangxi	0.265**	0.114	0.136	0.088	0.271*	0.06
2009	0.173	0.116	0.38***	0.107	−0.058	0.586
*Constant*	−0.789*	0.454	−2.249***	0.554	−1.896***	0
	N	8270	N	8270	N	8270
	LR chi2(27)	3376.57	LR chi2(27)	301.96	LR chi2(27)	272.87
	Prob > chi2	0	Prob > chi2	0	Prob > chi2	0
	Pseudo R2	0.5845	Pseudo R2	0.1016	Pseudo R2	0.1294

Note: Per capita household income is inflated to year 2009 using consumer price index. *** p < 0.01, ** p < 0.05, * p < 0.1.

Results of the probit regiression (Table 3) suggest that, *ceteris paribus*, the use of outpatient care was found to be associated with need factors and place of residence. Female, those who were with major illness or had been ill or injuried for the past 4 weeks were more likely to use outpatient care.

Folk doctor care was associated with people aged 30 and above, as well as people with major illness. It is also worth pointing out that those who were covered by the NCMS and other insurance were less likely to use folk doctor care compared with the uninsured.

In terms of preventive care, female, those with major and minor illness were more likely to use outpatient care. Income was significantly associated with the use of preventive care. Unskilled and agricultural workers were less likely to use preventive care compared with white collars and skill workers. People with no education, primary and secondary education, and high school education were less likely to use preventive care compared with people with university education or above. No significant association was observed in terms of the NCMS and preventive care use.

Those who participated in commercial insurance had a significantly lower level of outpatient use and a higher level of preventive care use compared with the uninsured. This may suggest that the rural Chinese had started to participate in commercial insurances on top of the NCMS to avoid potential financial risks and to improve access.

### Equity in health care use

Although the descriptive analysis and regression models showed some results of health inequity, the level of inequity and how it was driven by socioeconomic factors and health needs remains unclear. Table 4 provided need-adjusted and unadjusted health use by income quintiles. Table 5 showed the results of the Erreygers’s Concentration Index (EI). For each health care type, the table provided an index of socioeconomic inequity in use (EI), indicating the level of inequity of actual health use, and horizontal inequity (HI), indicating the level of inequity driven only by individual’s socioeconomic status. Confidence intervals were calculated using bootstrapping methods. The indices standardized by Probit Model are presented in Additional file 1 as a comparison.

**Table 4:** Health service use by income quintiles (linear probability model)

			**Poorest**	**2nd poorest**	**Middle**	**2nd richest**	**Richest**
Outpatient use	2004	Unadjusted	11.91%	10.01%	9.51%	11.91%	12.32%
		Need-adjusted	11.61%	10.72%	10.73%	12.42%	11.76%
	2009	Unadjusted	12.45%	10.97%	11.65%	9.97%	12.86%
		Need-adjusted	11.54%	11.57%	12.08%	10.67%	10.82%
Folk doctor use	2004	Unadjusted	3.91%	4.01%	3.90%	2.72%	2.01%
		Need-adjusted	3.60%	3.84%	3.76%	2.49%	1.70%
	2009	Unadjusted	6.09%	7.00%	4.20%	4.19%	3.60%
		Need-adjusted	5.77%	6.87%	4.09%	4.19%	3.22%
Preventive care use	2004	Unadjusted	1.50%	2.21%	2.30%	3.22%	5.72%
		Need-adjusted	1.49%	2.40%	2.51%	3.36%	5.69%
	2009	Unadjusted	1.99%	2.69%	3.69%	3.79%	5.29%
		Need-adjusted	2.03%	2.88%	3.90%	4.11%	5.25%

**Table 5:** Socioeconomic concentration indices by linear probability model (Erreyger’s concentration index)

		**2004**	**2009**
**Outpatient care**	EI	−0.0017	−0.0050
	Confidence Interval	(−0.021, 0.018)	(−0.027, 0.018)
	HI	0.0046	−0.0019
	Confidence Interval	(−0.009, 0.019)	(−0.017, 0.013)
**Folk doctor care**	EI	−0.0164	−0.0206
	Confidence Interval	(−0.028, -0.005)	(−0.037, -0.004)
	HI	−0.0154	−0.0192
	Confidence Interval	(−0.027, -0.004)	(−0.036, -0.003)
**Preventive care**	EI	0.0265	0.0222
	Confidence Interval	(0.015, 0.038)	(0.011, 0.033)
	HI	0.0268	0.0233
	Confidence Interval	(0.016, 0.038)	(0.012, 0.034)

Note: *EI* represents Inequity Indices for actual use, *HI* represents Horizontal Inequity. Confidence interval is set at 0.1 significance level.

Table 4 shows the prevalence of health care use by income quintiles with adjusted and unadjusted needs by years. Outpatient care was equally distributed among all income groups in 2004 and 2009. However, the use of folk doctor was more concentrated among the low income groups, and the use of preventive care was more concentrated among the high income groups, even after controlling for needs.

In terms of the inequity indices, a favouring-poor inequity was observed for outpatient care; however, both indices were not significant at 0.1 significant level. This means that there was not any inequity in term of outpatient use across income groups because the indices were not different from 0.

However, favouring-poor inequities were observed for folk doctor use. Both the EI and HI indices showed that the level of inequities had increased from 2004 to 2009. All indices were significant at 0.1 significant level. This means that the poor were more likely to visit folk doctor compared with the better-off, and the inequity effects had increased.

In terms of preventive care, a favouring-rich inequity was observed for both 2004 and 2009. However, it is worth pointing out that EI for preventive care was 0.026 in 2004 and 0.022 in 2009; HI were 0.027 in 2004 and 0.023 in 2009. The inequity effect was decreased, and preventive care was less concentrated among the better-off in 2009 compared with 2004.

### Decomposition analysis

**Figure 1:**
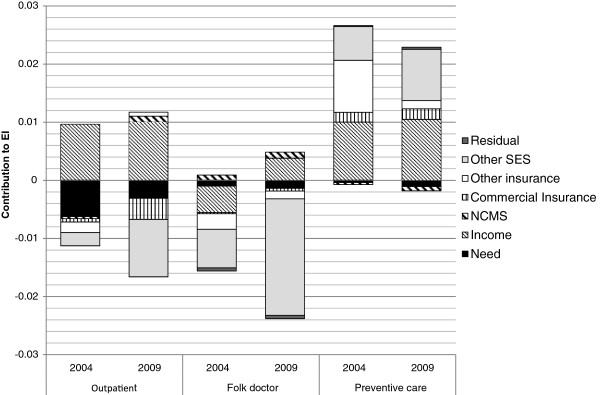
Components of Erreyger’s Concentration Indices in the probability of health service use (Linear Probability Model).

Figure 1 presents the results of the decomposition analysis, depicting the contribution of income-related health inequity from both need and socioeconomic factors.

Results from decomposition analysis confirm with the previous findings. In general, the use of health services was largely influenced by non-need/socioeconomic factors, especially for preventive care use and folk doctor use.

As the inequity indices for outpatient care were statistically insignificant. The decomposition analyses for outpatient care use were presented only as references.

In terms of folk doctor care, the inequity indices had increased from −0.016 in 2004 to −0.021 in 2009. Decomposition analysis showed favouring-poor inequities were mostly driven by non-need/socioeconomic factors. The NCMS reduced the observed inequity in folk doctor use because the NCMS was concentrated among the lower-income groups; however, the observed effect of the NCMS in reducing inequity was rather limited.

In terms of prevention care use, income, commercial insurance, and other socioeconomic factors were major contributors towards the total inequity. The use of preventive care was disproportionally concentrated among the rich. The NCMS contributed negatively to the observed income-related inequity in preventive care use, in other words, it reduced the level of inequity in preventive care use. However, the effect of the NCMS in reducing the inequity effects was still limited. In terms of commercial insurance, it contributed positively to the observed income-related inequity in preventive care use because higher income earners were both more likely to have commercial insurance and to use preventive care.

Additional file 2 presents the results for decomposition analysis using the Linear Probability Model. Additional file 3 presents the results by Probit Model. As expected, Probit models demonstrated similar results as Linear Probability Models.

## Discussion and conclusion

The study reveals a mixed picture in terms of the variation of health care utilisation and how the NCMS has influenced the level of inequity. Although the data used in this study represent only two points in time, it covers the whole period of the expansion of the NCMS from 2004 (before the national rollout of NCMS) and 2009 (after the expansion of NCMS across the rural China). The study has yielded some interesting findings. First, the study found that the level of inequity remained the same for outpatient care. In terms of preventive care, a pro-rich inequity was observed both in 2004 and 2009, but the level of inequity had decreased. However, an enlarged gap between the poor and the rich in terms of folk doctor use was observed. Decomposition analysis showed that the NCMS had helped to reduce income-related health inequity in folk doctor care and preventive care, but the contribution was rather limited. Commercial insurance had contributed towards the inequity in preventive care use; this suggested that the better-off were more likely to participate in commercial insurance and to use preventive care. Other socioeconomic factors including income had contributed positively to inequity in health use.

The findings were consistent with some of the previous research. Zhou et al. [16] suggested that inpatient care use was concentrated among the better-off, but the inequity indices decreased from 0.224 in 2003 to 0.115 in 2008. In terms of outpatient care use for mid-aged and elderly people, Wang et al. [15] found that in more affluent provinces like Zhejiang, outpatient use was concentrated among the better-off, while in provinces with low economic development, such as Gansu, use of health care was equally distributed across income groups. The study also suggested that this may be because of the difference in terms of health care provision and coverage of insurance between these two provinces. In terms of folk doctor care, the growing inequity between the rich and the poor is troubling, and such a problem is particularly severe for low income groups. Similar findings were demonstrated in studies conducted in other developing countries. These studies suggested that demand of lower social classes for care was highly price-elastic and usually exceeded that of the rich [50-52]. Hence, the poor were more likely to use more informal and less qualified providers, or resorted to self-treatment when they were ill [53].

To compare the level of inequity in health use of China with other countries, Van de Poel et al. [54] showed that the Erregyers’ Concentration Indices of all health care use was 0.1 in India, 0.018 in Malaysia and 0.018 in Bangladesh, which seemed comparable with the indices from China. This suggested that, in a comparative sense, China was in a similar level of equity in health utilisation as other low- and middle-income countries.

The study has a few policy implications. The extension of the NCMS coverage reduces inequitable access in formal care, but does not eliminate them. One important constraint of the NCMS is the low reimbursement rate and the high co-payment at visit. Reported average reimbursement rate for outpatient care under the NCMS was only approximately 10% [10]; it is argued that even though out-of-pocket payments for outpatient care may be easy to cope with in a short term, a large amount of outpatient costs in aggregate may still be excessively high from a social standpoint and may have substantial effects on household [9]. Similarly, the use of preventive care is unequally distributed and related to the unequal distribution of income level. A more comprehensive coverage in terms of outpatient care and preventive care is needed because outpatient care is the most commonly used for effective and efficient treatment for many health problems, especially chronic diseases, and preventive care is equally important in terms of allowing for early detection of diseases.

The NCMS aimed to achieve equity in the contribution through co-payments regardless of income levels of the participants; however, among the NCMS participants, there existed a wide gap in financial status. Low income participants are already burdened with a premium, while substantial co-payments due to the limited coverage further aggravate HI in health care access [55]. A possible solution is to implement well-designed and regulated health insurance with comprehensive coverage to provide the low income participants with better financial protection. Successful examples include - Universal Coverage scheme of Thailand and *Seguro Popular* of Mexico for the poor and uninsured [56-58].

It is also worth mentioning that more and more rural Chinese, especially the better-off, are seeking for financial protection from participating in commercial insurance. The NCMS with the objective of protecting the vulnerable groups from financial barriers to care had a slightly favouringpoor effect, whereas commercial insurance contributed to the favouring-rich distribution of health use. The movements towards a more generous benefit package of the public funded insurance - the NCMS - is crucial in improving access to care and to better align health use with need. For services not being covered by the NCMS, as exemplified by the study results, resorting to commercial insurance to improve equity in access might be one option for the rural Chinese.

The study has a few limitations. The first concern is the dataset. The dataset used is probably by far the most comprehensive ever used in studying health inequality in the Chinese context; however, only nine provinces are included. Most of these provinces are situated in the eastern and coastal part of China, where the levels of economic development are high. Hence, any further generalization should be made with caution. As all the survey information is self-reported, this can be biased because of problems in reporting (e.g. inaccurate recall, misreporting). However, these are the limitations of using self-assessed morbidity measurements in the absence of other possible objective variables, such as biomarker. Second, the difference between what is officially called informal care and what happens in practice needs further refinement in future studies. In this dataset, all informal care providers are evaluated at the same standard, and are specified as “folk doctor care”; however, it is possible that folk doctor use may relate to the use of traditional Chinese medicines and healer, which are widely accepted and even recommended in some medical settings [59-61]. Therefore, the dataset needs further refinement in the definition of folk doctor care in order to make inference on equity of use.

## Abbreviations

CHNS: China health and nutrition survey; LPM: Linear probability model; EI: Erreygers’s concentration index; HI: Horizontal inequity.

## Competing interests

The author declared that she has no competing interest.

## Supplementary Material

Additional file 1 Socioeconomic Concentration Indices by Probit Model (Erreygers’s Concentration Index). Click here for file

Additional file 2 Decomposition results by Linear Probability Model (Components of Erreyger’s Concentration Indices). Click here for file

Additional file 3 Decomposition results by Probit Model (Components of Erreyger’s Concentration Indices). Click here for file
