# Supplementary material for: China’s new cooperative medical scheme and equity in access to health care: evidence from a longitudinal household survey
Source: Int J Equity Health. 2013 Mar 23;12:20. doi: 10.1186/1475-9276-12-20 (PMC3616826; doi:10.1186/1475-9276-12-20)
Supplement: Additional file 1 — Socioeconomic Concentration Indices by Probit Model (Erreygers’s Concentration Index). [file 1475-9276-12-20-S1.doc]

Appendix 1. Socioeconomic Concentration Indices by Probit Model ( Erreygers’s Concentration Index)

|  |  | **2004** | **2009** |
| --- | --- | --- | --- |
| **Outpatient care** | EI | -0.0017 | -0.0050 |
|  | Confidence Interval | (-0.021, 0.018) | (-0.027, 0.018) |
|  | HI | 0.0054 | -0.0007 |
|  | Confidence Interval | (-0.009, 0.019) | (-0.023, 0.022) |
| **Folk doctor care** | EI | -0.0164 | -0.0206 |
|  | Confidence Interval | (-0.028, -0.005) | (-0.037, -0.004) |
|  | HI | -0.0157 | -0.0192 |
|  | Confidence Interval | (-0.027, -0.005) | (-0.036, -0.003) |
| **Preventive care** | EI | 0.0265 | 0.0222 |
|  | Confidence Interval | (0.015, 0.038) | (0.011, 0.033) |
|  | HI | 0.0267 | 0.0230 |
|  | Confidence Interval | (0.016, 0.038) | (0.012, 0.034) |

Note: EI represents Inequity Indices for actual use, HI represents Horizontal Inequity. Confidence interval is set at 0.1 significance level.
