# Supplementary material for: China’s new cooperative medical scheme and equity in access to health care: evidence from a longitudinal household survey
Source: Int J Equity Health. 2013 Mar 23;12:20. doi: 10.1186/1475-9276-12-20 (PMC3616826; doi:10.1186/1475-9276-12-20)
Supplement: Additional file 2 — Decomposition results by Linear Probability Model (Components of Erreyger’s Concentration Indices). [file 1475-9276-12-20-S2.doc]

Additional file 2. Decomposition results by Linear Probability Model (Components of Erreyger’s Concentration Indices)

|  | **Outpatient** | | **Folk doctor** | | **Preventive care** | |
| --- | --- | --- | --- | --- | --- | --- |
|  | **2004** | **2009** | **2004** | **2009** | **2004** | **2009** |
| 30-44 | 0.0005 | -0.0013 | 0.0027 | 0.0021 | -0.0026 | 0.0013 |
| 45-64 | -0.0010 | 0.0001 | -0.0011 | -0.0002 | 0.0012 | 0.0000 |
| 65 and above | -0.0020 | 0.0018 | -0.0021 | -0.0032 | 0.0012 | -0.0014 |
| Gender (1 = male) | -0.0002 | -0.0003 | 0.0000 | -0.0002 | -0.0003 | -0.0001 |
| No symptoms | -0.0011 | 0.0002 | -0.0002 | 0.0002 | -0.0001 | 0.0000 |
| Minor Illness | 0.0000 | 0.0001 | 0.0001 | -0.0001 | 0.0000 | -0.0004 |
| 4 week illness | -0.0025 | -0.0036 | -0.0004 | -0.0001 | 0.0000 | -0.0005 |
| Per capita income (lg) | 0.0096 | 0.0100 | -0.0045 | 0.0038 | 0.0100 | 0.0105 |
| NCMS | -0.0004 | 0.0010 | 0.0009 | 0.0011 | -0.0004 | -0.0008 |
| Commercial insurance | -0.0006 | -0.0036 | -0.0002 | -0.0005 | 0.0017 | 0.0018 |
| Other insurance | -0.0018 | 0.0007 | -0.0027 | -0.0013 | 0.0089 | 0.0014 |
| Marital Status (1 = married) | -0.0002 | 0.0000 | -0.0001 | 0.0010 | 0.0002 | -0.0001 |
| Unskilled and agriculture | 0.0025 | 0.0000 | 0.0010 | -0.0007 | 0.0009 | 0.0007 |
| Other job | -0.0002 | 0.0000 | 0.0005 | -0.0004 | -0.0003 | -0.0011 |
| Unemployed | 0.0007 | 0.0021 | 0.0001 | -0.0040 | -0.0003 | 0.0020 |
| No edu | 0.0087 | -0.0141 | -0.0041 | -0.0005 | 0.0160 | 0.0107 |
| Pri and sec edu | -0.0004 | 0.0029 | 0.0002 | -0.0003 | -0.0011 | -0.0024 |
| High school | -0.0063 | 0.0045 | 0.0033 | -0.0010 | -0.0123 | -0.0053 |
| Province Liaoning | -0.0025 | -0.0004 | -0.0034 | -0.0004 | -0.0002 | 0.0001 |
| Province Heilongjiang | -0.0003 | -0.0002 | -0.0027 | -0.0003 | -0.0001 | -0.0001 |
| Province Jiangsu | -0.0031 | 0.0024 | -0.0026 | -0.0042 | 0.0019 | 0.0063 |
| Province Shandong | 0.0006 | -0.0001 | -0.0010 | 0.0005 | 0.0013 | 0.0001 |
| Province Henan | -0.0011 | -0.0032 | 0.0003 | -0.0035 | 0.0000 | -0.0008 |
| Province Hubei | -0.0004 | -0.0003 | 0.0008 | -0.0011 | -0.0005 | 0.0005 |
| Province Hunan | 0.0002 | -0.0005 | -0.0001 | -0.0003 | 0.0002 | 0.0000 |
| Province Guangxi | -0.0004 | -0.0030 | 0.0012 | -0.0047 | 0.0000 | -0.0017 |
| Residual | 0.0000 | 0.0000 | -0.0006 | -0.0006 | 0.0002 | 0.0004 |
